# Supplementary material for: Disease-associated mutations in Niemann-Pick type C1 alter ER calcium signaling and neuronal plasticity
Source: J Cell Biol. 2019 Oct 10;218(12):4141–56. doi: 10.1083/jcb.201903018 (PMC6891088; doi:10.1083/jcb.201903018)
Supplement: Supplemental Materials (PDF) [file JCB_201903018_sm.pdf]

Supplemental material

Tiscione et al., https://doi.org/10.1083/jcb.201903018

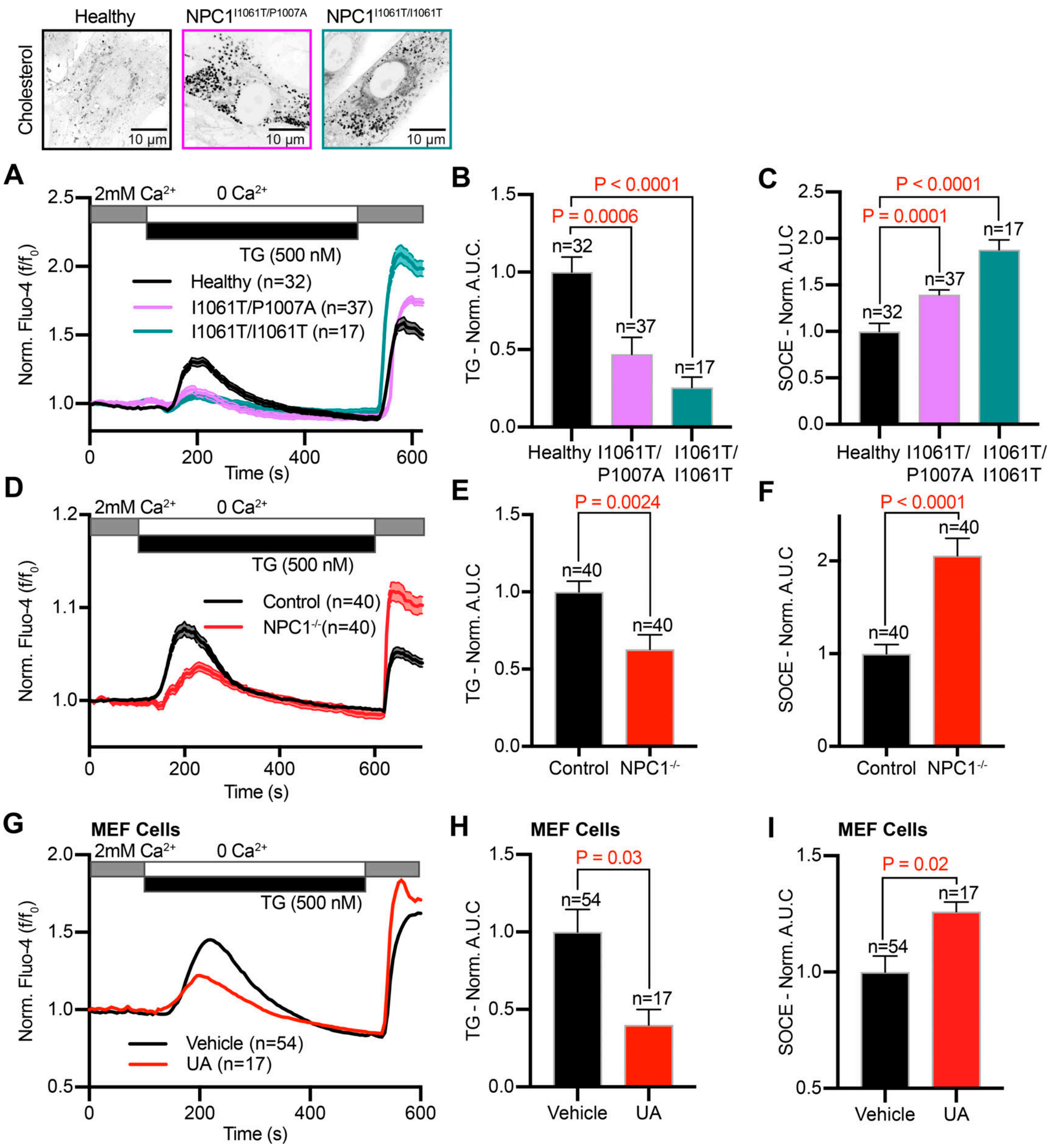

Figure S1. **Ca^{2+} signaling is altered following NPC1 disease mutations, NPC1$^{-/-}$, or NPC1 inhibition. (A)** Top: Filipin staining in healthy, NPC1$^{I1061T/I1061T}$, and NPC1$^{I1061T/P1007A}$ fibroblasts. Bottom: Averaged time-series from Fluo-4–loaded healthy, NPC1$^{I1061T/I1061T}$, NPC1$^{I1061T/P1007A}$ fibroblasts following addition of TG (500 nM) in the absence or presence of 2 mM external Ca^{2+}. **(B and C)** Quantification of TG and SOCE AUC for each cell type. **(D)** Averaged time series from Fluo-4–loaded control and NPC1$^{-/-}$ CHO cells. **(E and F)** same analysis as B and C. **(G)** Averaged time series from Fluo-4–loaded control (vehicle-treated) and UA-treated (500 nM overnight treatment) MEFs. **(H and I)** Same analysis as B and C. TG, TG 500 nM. Scale bars represent 10 µm. P values shown are calculated from parametric Student's *t* tests. *n* represents the total number of cells analyzed for each experimental condition. Error bars represent the standard error of the mean.

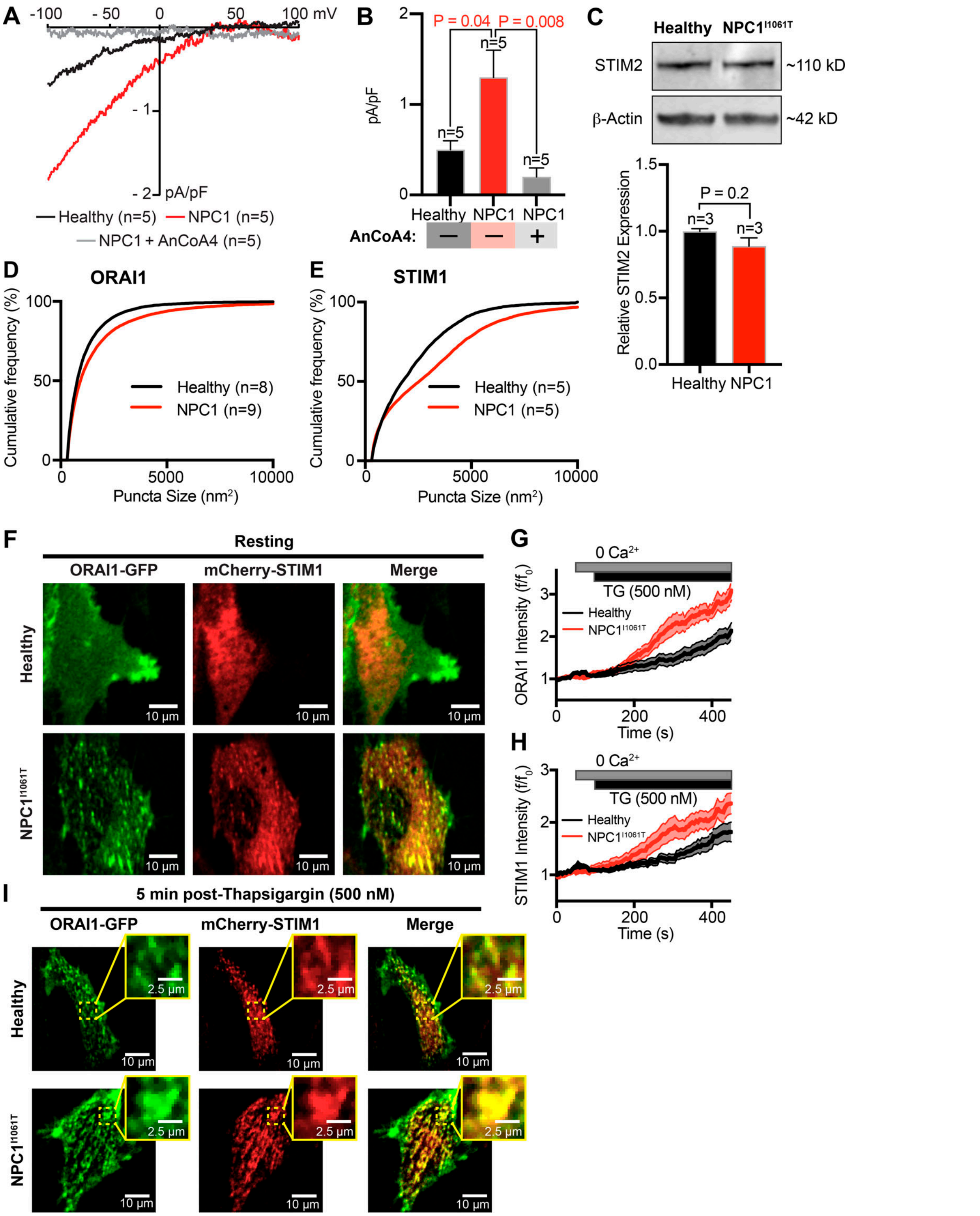

Figure S2. **$NPC1^{I1061T}$ fibroblasts have elevated I_{CRAC} and enhanced ORAI1 STIM1 puncta formation and kinetics. (A)** Left: Representative traces of I_{CRAC} currents recorded from healthy (black) and $NPC1^{I1061T}$ fibroblasts (red) and $NPC1^{I1061T}$ fibroblasts treated with ORAI1 inhibitor AnCoA4 (gray). **(B)** Quantification of current density with and without treatment with ORAI1 inhibitor. **(C)** Top: Representative Western blot showing STIM2 in healthy fibroblasts and $NPC1^{I1061T}$ fibroblasts. Bottom: Quantification of the differential protein expression, normalized to β-actin. **(D)** Cumulative frequency analyses for ORAI1 puncta from healthy (black) and $NPC1^{I1061T}$ fibroblasts (red). **(E)** Same as D, only STIM1. **(F)** Representative live confocal images of healthy and $NPC1^{I1061T}$ fibroblasts expressing ORAI1-GFP and mCherry-STIM1; shown individually and merged. **(G and H)** Time courses of ORAI1-GFP (healthy n = 9, $NPC1^{I1061T}$ n = 7) and mCherry-STIM1 (healthy n = 9, $NPC1^{I1061T}$ n = 7) puncta intensities following addition of TG in a Ca^{2+}-free external solution. **(I)** Representative live confocal images of ORAI1-GFP, mCherry-STIM1, or merged, following a 5-min treatment with TG in healthy and $NPC1^{I1061T}$ fibroblasts. P values shown are calculated from parametric Student's t tests. n represents the total number of cells analyzed for each experimental condition. Error bars represent the standard error of the mean.

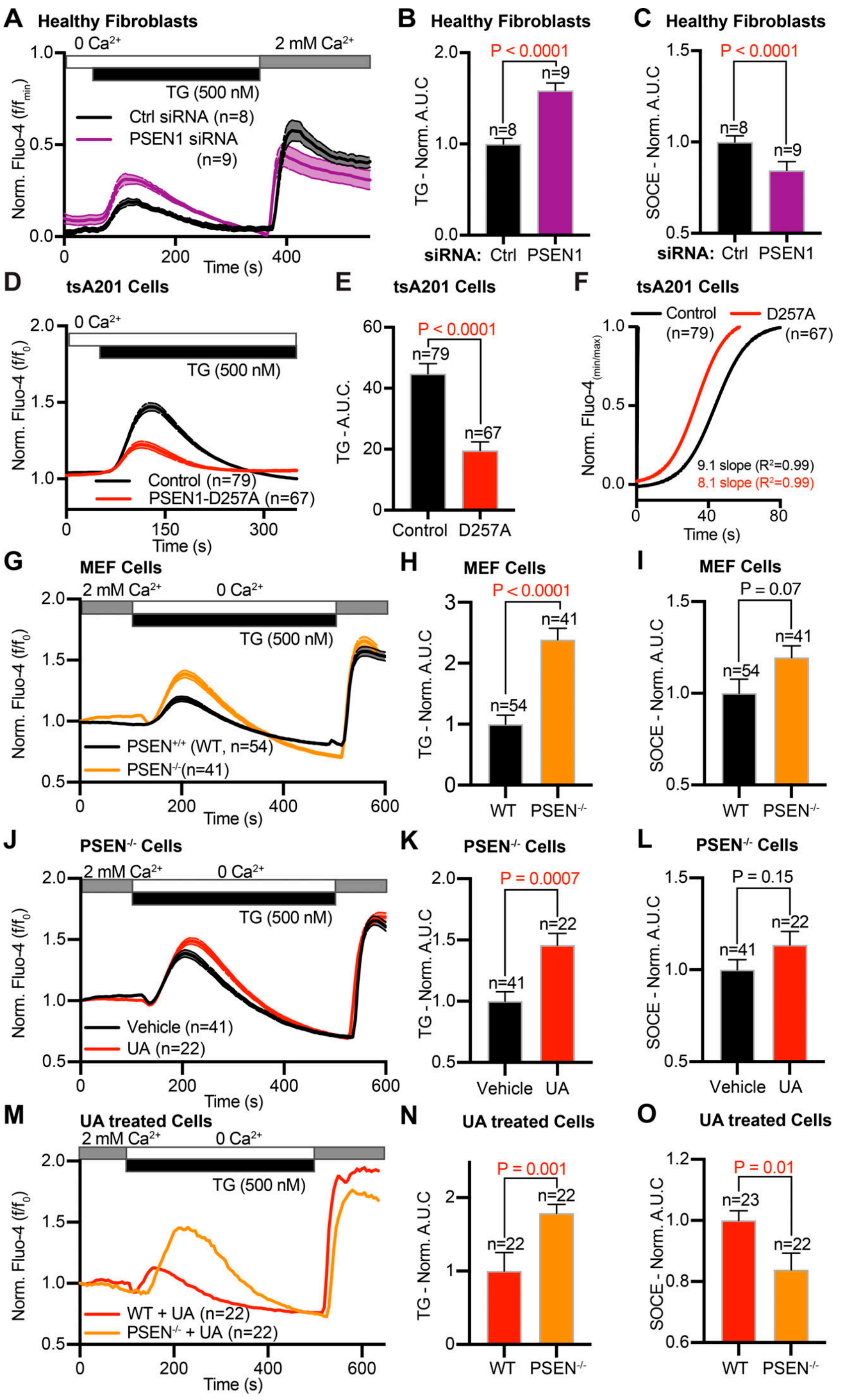

Figure S3. **PSEN1 expression alters ER Ca^{2+} Levels. (A)** Averaged Fluo-4 responses to TG from healthy fibroblasts treated with control (black) or PSEN1 siRNA (purple). **(B)** Quantification of TG AUC from traces in A. **(C)** Quantification of SOCE AUC from traces in A. **(D)** Normalized Fluo-4 TG responses from control (black) tsA201 cells or those overexpressing PSEN1 D257A (red). **(E)** Quantification of TG AUC. **(F)** TG slope analysis from traces in D. **(G–I)** Same design as A–C except with control (black) or PSEN1$^{-/-}$ (orange) MEFs. **(J–L)** Same design as A–C except with PSEN1$^{-/-}$ cells (black) or PSEN1$^{-/-}$ cells treated with UA for 24 h (red). **(M–O)** Same design as A–C except with control MEFs treated with UA for 24 h (red) or PSEN1$^{-/-}$ cells treated with UA for 24 h (orange). P values shown are calculated from parametric Student's *t* tests. *n* represents the total number of cells analyzed for each experimental condition. Error bars represent the standard error of the mean.

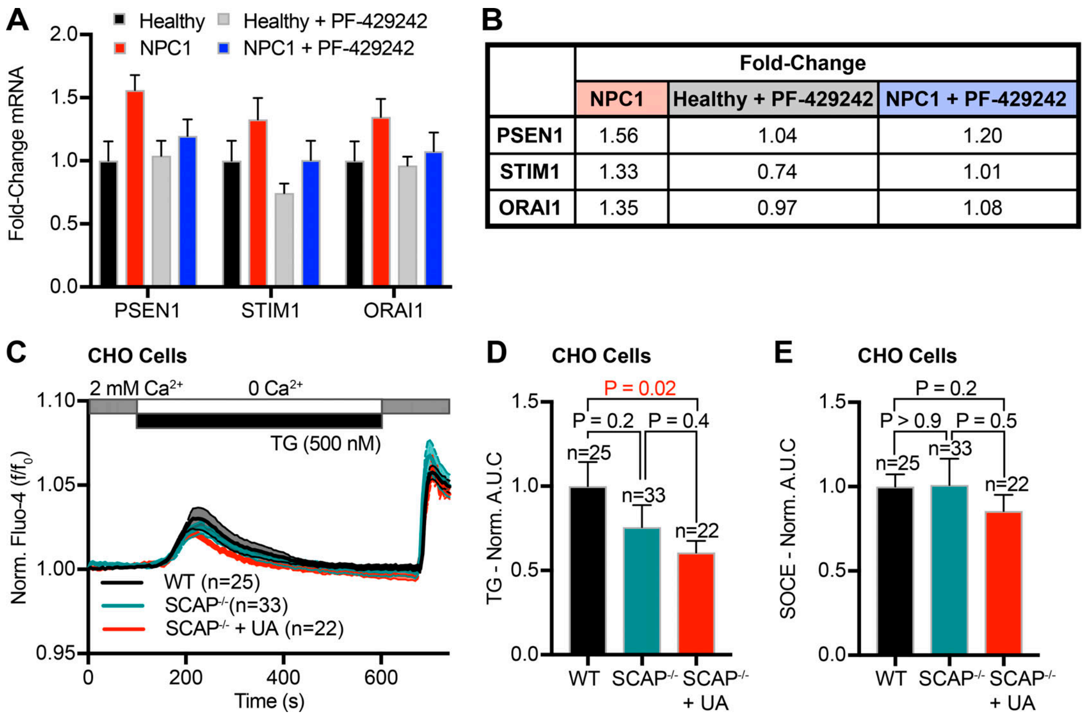

	Fold-Change		
	NPC1	Healthy + PF-429242	NPC1 + PF-429242
PSEN1	1.56	1.04	1.20
STIM1	1.33	0.74	1.01
ORAI1	1.35	0.97	1.08

Figure S4. **mRNA levels of key Ca^{2+} handling proteins are regulated by SREBP. (A)** Fold change in mRNA levels of PSEN1, STIM1, and ORAI from healthy cells (black), NPC1^{I1061T} cells (red), healthy cells treated with PF-429242 for 24 h (gray), and NPC1^{I1061T} cells treated with PF-429242 for 24 h (blue; *n* = 6 for all mRNA and conditions). **(B)** Tabulation of results in A. **(C)** Averaged, normalized Fluo-4 TG responses from WT cells (black, WT), SCAP$^{-/-}$ cells (green), and SCAP$^{-/-}$ cells treated with UA for 24 h (red). **(D and E)** Quantification of TG AUC and SOCE AUC for each condition, respectively. P values shown are calculated from parametric Student's *t* tests. *n* represents the total number of cells analyzed for each experimental condition. Error bars represent the standard error of the mean.

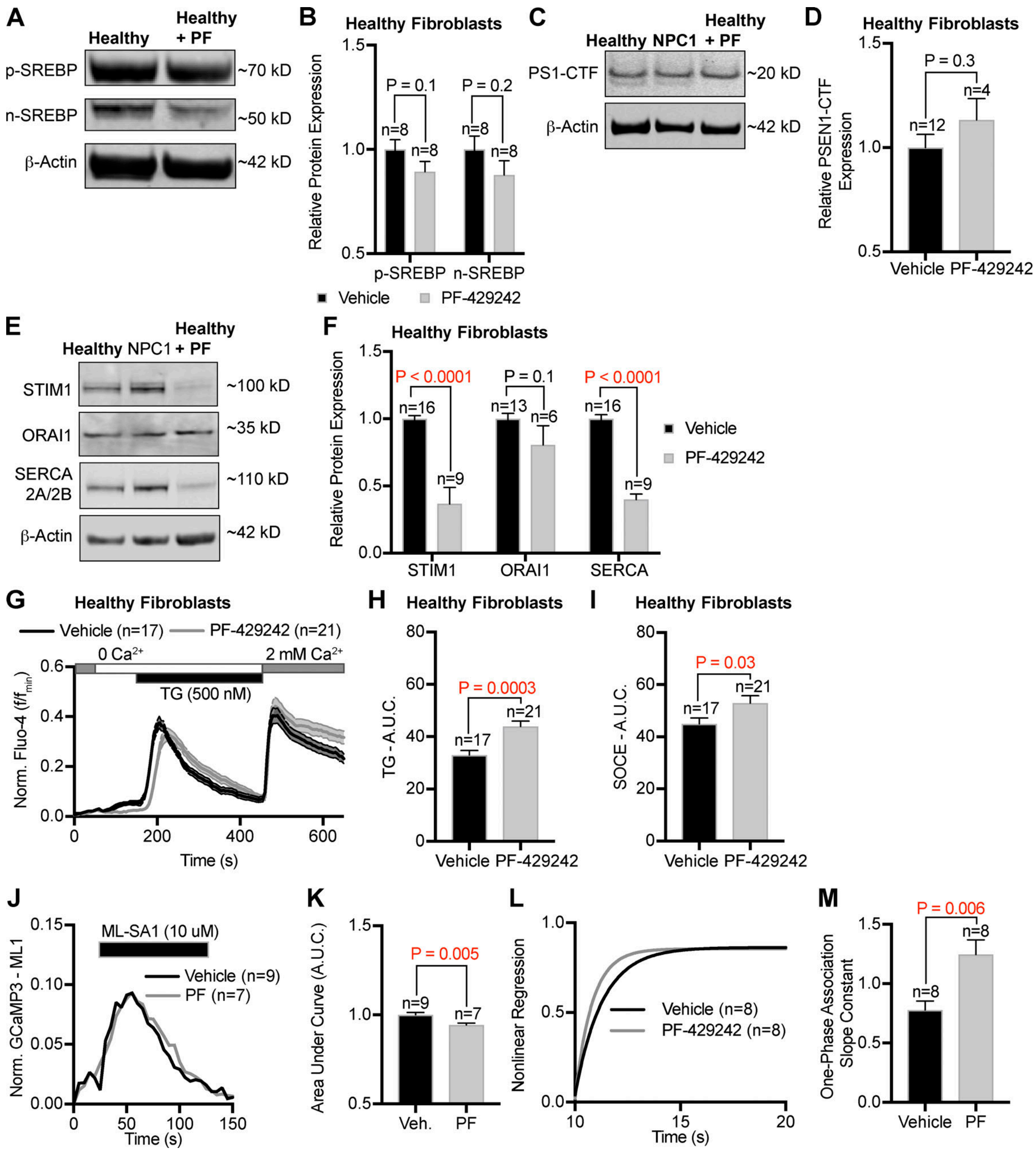

Figure S5. **Effect of PF-429242 on Ca^{2+} dynamics and lysosomal function in control cells. (A)** Representative inactive SREBP (p-SREBP) and active SREBP (n-SREBP) Western blots in healthy fibroblasts treated 24 h with vehicle or SREBP inhibitor, PF-429242. **(B)** Quantification of differential SREBP expression, normalized to β-actin. **(C)** Representative Western blot showing PSEN1-CTF protein in healthy fibroblasts treated 24 h with vehicle or PF-429242. **(D)** Quantification of the differential PSEN1-CTF expression, normalized to β-actin. **(E)** Representative Western blot showing SERCA, STIM1, and ORAI1 in healthy fibroblasts treated 24 h with vehicle or PF-429242. **(F)** Quantification of the differential protein expression, normalized to β-Actin. **(G)** Normalized Fluo-4 time series of healthy fibroblasts treated 24 h with vehicle (black) or PF-429242 (gray). **(H)** Quantification of the AUC during TG. **(I)** Quantification of the AUC during SOCE. **(J)** Representative, normalized changes in GCAMP3-ML1 intensity from tsA201 cells treated with a vehicle control (black) or PF-429242 (gray). **(K)** Quantification of the AUC following addition of ML-SA1. **(L)** One-phase association curves fitted to the average traces showing fluorescent recovery of lyso-pHoenix following photoactivation. **(M)** Quantitative comparison of the slope constants (K value) in the nonlinear regression curves. P values shown are calculated from parametric Student's *t* tests. *n* represents the total number of cells analyzed for each experimental condition. Error bars represent the standard error of the mean.
